# Virtual nutrition consultation: what can we learn from the COVID-19 pandemic?

**DOI:** 10.1017/S1368980021000148

**Published:** 2021-01-13

**Authors:** Vered Kaufman-Shriqui, Shiri Sherf-Dagan, Mona Boaz, Ruth Birk

**Affiliations:** Department of Nutrition Sciences, School of Health Sciences, Ariel University, P.O. Box 3, Ariel, Israel

**Keywords:** Coronavirus, COVID-19, Dietitians, Nutrition, Nutrition survey, Telemedicine

## Abstract

**Objective:**

To investigate the extent, quality and challenges of dietetic counselling during the pandemic.

**Design:**

A cross-sectional online thirty-six-item Google Survey. The survey queried demographics and information on usage and perceived telemedicine quality.

**Setting:**

The survey was distributed to Israeli Dietetic Association (ATID) mailing list between 31 March and 5 May 2020.

**Participants:**

Clinical dietitians, members of ATID, who consented to participated in the survey.

**Results:**

Three hundred dietitians (12 % of ATID members; 95 % women; mean age 4·41 (sd 10·2) years) replied to the survey. Most dietitians reported a significant ∼30 % decrease in work hours due to the pandemic. The most prevalent form of alternative nutrition counselling (ANC) was over the phone (72 %); 53·5 % used online platforms. Nearly 45 % had no former ANC experience. Both ANC formats were reported inferior to face-to-face nutritional consultation (consultation quality median scores 8 and 7, on a 1–10 scale, for online and phone, respectively). ANC difficulties on either phone or online platforms were technical (56 and 47 %, respectively), lack of anthropometric measurements (28 and 25 %, respectively) and interpersonal communication (19 and 14·6 %, respectively). Older age and former phone counselling experience were associated with higher quality scores, respectively (OR = 1·046, 95 % CI 1·01, 1·08, *P* = 0·005), (95 % CI 1·38, 4·52, *P* = 0·02). Those who continued to work full time had five-time greater odds for a higher quality score using online platforms (OR = 5·33, 95 % CI 1·091, 14·89, *P* = 0·001).

**Conclusions:**

Our findings suggest telemedicine holds considerable promise for dietary consultation; however, additional tools and training are needed to optimise remote ANC, especially in light of potential crisis-induced lockdown.

The coronavirus disease 2019 (COVID-19), an infectious disease caused by a novel coronavirus SARS-COV-2, was declared an epidemic by the WHO and became a major global human health threat^(1)^. One of the factors influencing the outcome of patients with COVID-19 is nutrition status^(2)^. Several nutrition-related health issues are of major concern during, and as a consequence of, the COVID-19 outbreak. Self-isolation policies that were enforced during this outbreak have been related to an increase in irregular eating patterns, poor diet quality, sedentary behaviours, poor sleep quality, weight gain, emotional disturbance and mental health disorders^(1)^. Furthermore, the global economic changes due to the COVID-19 outbreak may introduce food insecurity to new populations and worsen existing food insecurity among those already vulnerable prior to the outbreak^(1)^. Thus, along with the needed immediate healthcare response, it is also essential to consider the long-term health consequences of this pandemic^(3)^.

It is well established that nutrition plays a role in immune system function^(1,4)^; however, during the COVID-19 outbreak, inaccurate reports about nutrition frequently appeared on social media including misleading messages of single food, vitamin or herb promising cure or prevention of the disease^(1)^. It is critical that dietitians provide reliable and evidence-based nutrition information during this challenging period.

COVID-19 was identified in Israel during mid-March 2020. As of May 2020, there have been 16 589 cases reported in the population of approximately 9 million, with a relatively low fatality rate of 31/1 000 000^(5)^. In an attempt to control disease spread, the government has enforced strategies of self-isolation, quarantine (which began at 25 March 2020), screening, mitigation and/or suppression.

During this period, ambulatory health services were closed, and elective procedures were postponed^(1,3)^. To permit the ongoing provision of healthcare services despite the lockdown, many health professionals began using various virtual clinical consultation formats^(3)^, rapidly increasing telemedicine usage in various clinical fields^(6–10)^. While telehealth refers to broader scope of remote healthcare services, telemedicine refers specifically to remote clinical services only^(3)^. However, the extent to which dietitians adopted virtual consultation techniques during the COVID-19 outbreak is unknown.

Therefore, the purpose of the current study was to assess how the COVID-19 outbreak affected the professional practices of dietitians, including the extent to which dietitians have used various virtual consultations platforms, and the overall quality and utility of these modalities.

## Methods

### Overall study design and plan

The respondents to this cross-sectional survey were a convenience sample of registered dietitians (RD). Investigators sent an online survey to the Israeli Dietetic Association (ATID), which posted the survey using their electronic mailing list. The study was conducted online using a Google Survey platform. Data collection was performed from 31 March 31 to 5 May, a period of time during which the Israeli population was under wide-scale self-isolation.

### Ethics

The study was approved by the Institutional Ethics Committee. Each participant provided an electronic informed consent prior to responding to the survey.

### Survey development

The survey included thirty-six questions, featuring a questionnaire to assess participant perceptions and experiences of telemedicine using the phone and/or online platforms^(11)^. The questions selected assessed the domains of overall quality, technical quality, clinical quality and organisational difficulties (Likert scale 1–10) and additional questions on technical difficulties and future use (Likert scale 1–4). The survey also queried the demographic characteristics. The questionnaire was translated from English to Hebrew by a native English speaker who was also fluent in Hebrew. Once translated, the translation was back-translated to Hebrew by an individual bilingual in both the English language and Hebrew, thus validating the translation. Participants replied to the survey in Hebrew. Additionally, the survey questions and usability of the online platform were referred to a focus group of potential participants to assess readability and clarity; minor amendments were made following their feedback. Survey participants were asked to indicate both their work setting and the weekly number of hours of work and indicate if and how they continued to work during the COVID-19 outbreak. Respondents were asked to compare the quality of phone or online platforms to face-to-face counselling. Reply options were equal, superior or inferior to face-to-face counselling. Participants who indicated that they used both phone and online platforms were referred to an additional question: ‘How would you evaluate the overall professional quality of phone compared to online nutrition counseling?’ with three reply options: similar quality; online counselling is of higher quality or phone counselling is of higher quality. An open question asked participants to describe the difficulties that they experienced during counselling sessions; this information was thematically analysed by two dietitians who reached a consensus on the main topics. Additionally, participants used a ten-point Likert scale to estimate client comfort level during remote counselling. The final version of the questionnaire was reviewed by ten RD, providing expert validity.

### Data analysis

SPSS v. 25·0 (IBM Inc.) was used for all statistical analyses. Distributions of continuous variables were assessed for normality using the Kolmogorov–Smirnov test; thus, these are described using mean and SD. Continuous data had distributions significantly deviating from normal, so they are described as median (interquartile range). Categorical variables such as the proportion of participants with a given response were described using frequency counts and expressed as *n* (%). Associations between socio-demographic and occupational characteristics of dietitians and the overall quality of phone and online counselling were examined using logistic regression models with stepwise variable selection. The independent variables associated with the overall quality variable at a significance level of *P* ≤ 0·10 were considered for inclusion in multivariate models. Two separate models were developed, and the median value of the quality score at each type of consultation was assigned as the cutoff value. All tests are two-sided and considered significant at *P* < 0·05.

## Results

### The survey participants

Of 2500 registered members of ATID, 300 individuals replied (12 % of members). Table 1 describes the survey participants. The majority of participants were female, and their mean age was 4·41 (sd 10·2) years. The areas of study for the highest degree of education included nutrition (71·3 %), public health (15·0 %), management (5·4 %), medical sciences (3·0 %) and biology and biochemistry or chemistry (3·0 %). Additionally, 2·3 % of respondents indicated that their highest degree was in another field not specified. More than 40 % of survey participants held advanced degrees. Most participants indicated two fields of nutrition specialty or more, and the majority of dietitians included weight loss as their field of expertise (54·6 %).

**Table 1:** Characteristics of survey participants

Characteristics	Result (*n* 300)	
	*n*	%
Age, years		
Mean	4·41	
sd	10·2	
Sex (female)	285	95
Highest level of education		
BSc, BA	160	53·3
MSc, MA, MPH, MBA	121	40·3
PhD/MD	17	5·7
Other	2	0·7
Field of highest degree obtained		
Nutrition	214	71·3
Public Health/Epidemiology	45	15·0
Business Management/Health Systems Management	16	5·4
Biology/Biochemistry/Chemistry	9	3·0
Medical Sciences	9	3·0
Other	7	2·3
Employment setting*		
Public hospital	100	33·3
Private hospital	12	4·0
Health maintenance organisation (HMO)	94	31·3
Long-term care facility/rehabilitation facility Privately owned institution/clinic	11	3·7
Self-owned clinic	41	13·7
Private or chain clinic	18	6·0
Gym/fitness centre	14	4·7
Other	6	2·0
Years of professional experience		
Mean	14·1	
sd	10·6	
Field of expertise in nutrition counselling†		
Overweight/obesity/weight loss	164	54·6
CVD	5	1·7
Diabetes	29	9·7
Nephrology	10	3·3
Oncology	4	1·3
Gastrointestinal	15	5·0
Paediatrics (aged < 6 years)	18	6·0
Bariatric surgeries	8	2·7
Eating disorders	14	4·7
Sports nutrition	4	1·3
No area of clinical specialisation	18	6·0
Other	11	3·7
Number of hours/week performing nutrition counselling (prior to the COVID-19 outbreak)		
Mean	25·1	
sd	12·7	
Number of hours/week performing nutrition counselling (since the COVID-19 outbreak)		
Mean	17·1	
sd	10·7	
Work capacity after the onset of the COVID-19		
Full time	71	23·7
Part time	200	66·6
Have not worked as a dietitian since the outbreak	8	2·7
Unemployed since the outbreak	21	7·0

BSc, Bachelor of Science; BA, Bachelor of Arts; MSc, Master of Science; MA, Master of Arts; MPH, Master of Public Health; MBA, Master of Business Administration; Ph.D., Doctor of Philosophy; MD, Medical Doctor.

* Where the employed provided the option of multiple answers; 40·0 % indicated a single location of employment; however, 43·0 % of respondents indicated two locations of employment, and 15·7 % of respondents indicated three locations of employment, 1·3 % were missing information on employment setting.

** Where the field of expertise provided the option of multiple answers; 27·3 % indicated a single field of specialty; however, 28·0 % of respondents indicated two fields of specialty, 21·0 % of respondents indicated three fields of specialty, 12·3 % of respondents indicated four areas of specialty and the remaining 11·3 % indicated five areas of specialty.

The most frequently reported employment settings included hospital and outpatient clinics in health maintenance organisations. However, most participants indicated more than a single employment setting. The average number of weekly nutrition counselling sessions was 25·1 (sd 12·7) h prior to the outbreak. This decreased by 32 % during the outbreak, with the majority of the study participants (66·6 %) reporting working only part time since the outbreak.

### Characteristics of nutrition counselling

The majority of the participants continued to perform nutrition counselling during the shutdown to a certain extent (72·0 %). Table 2 describes the characteristics of nutrition counselling during the study period. Types of mixed counselling were frequent, with 24·7 % who performed either online consultation or phone consultation (while not meeting patients in person) and 6 % who met clients in-person, counselled over the phone or used online counselling only. The greatest proportion of respondents who performed online counselling used the ZOOM video platform (49 %), followed by WhatsApp (33 %) and Skype (14 %). An additional 4 % used Facebook Messenger, Google Meet, Microsoft Teams and Unicko.

**Table 2:** Characteristics of nutrition counselling since the COVID-19 outbreak

Characteristics	Result	
	*n*	%
The setting of nutrition counselling after the COVID-19 outbreak		
Usual nutrition counselling (face-to-face, in-person)	10	3·3
Usual nutrition counselling and phone nutrition counselling	59	19·7
Only phone nutrition counselling	62	20·7
Usual nutrition counselling and online nutrition counselling	20	6·7
Only online nutrition counselling	24	8·0
Only phone and online nutrition counselling	74	24·7
Usual nutrition counselling as well as phone and online nutrition counselling	18	6·0
Changes in client characteristics during the COVID-19 outbreak		
No changes in client characteristics	172	63·8
Younger clients	34	12·6
Older clients	9	3·3
More women	13	4·8
More men	3	1·1
Other	39	14·4
Changes in the type of nutrition counselling referrals (%)		
No change	113	43·9
Type of change in nutrition counselling referrals		
More referrals for weight loss/weight maintenance	82	48·5
More referrals for diabetes control	36	21·3
More referrals for CVD	4	2·4
More referrals for the paediatric nutrition	14	8·3
More referrals for eating disorders	13	7·7
More referrals for gastrointestinal problems	11	6·5
Other*	62	36·7

* The reply for the category ‘other’ included: no referrals at all for sport nutrition, hardly any new clients, just returning referrals and overall decrease in referrals.

Differences in the type of referrals were noted among 56·1 % of respondents; the majority of which (48·5 %) were identified as an increase in referrals for weight loss/weight maintenance. An additional 21·3 % reported more referrals for diabetes control, and 36·7 % reported other changes, among them a decrease in referrals overall, fewer new referrals and no referrals for sports nutrition.

### Nutrition consultation over the phone and by using online platforms

Characteristics of nutrition consultation over the phone and when using online video platforms are presented in Table 3. The majority (57·0 %) of respondents indicated they had no previous experience in performing consultation over the phone prior to the COVID-19 pandemic, while 18·2 % had little experience and 38·8 % had previous experience. More than half (65·4 %) indicated that phone consultation was inferior to the usual face-to-face consultation, while 25·2 % found the two techniques similar. Only 3·3 % found phone counselling superior to face-to-face counselling. The most frequently reported difficulties in using phone counselling included technical difficulties (56·2 %), followed by a lack of anthropometric measurements (25·2 %), interpersonal or communication difficulties (14·6 %) and difficulties stemming from conducting the session in the home environment (2·4 %).

**Table 3:** Characteristics of quality of telemedicine using the phone and online platforms

Question/response	Result			
	*n*	%	Median	Interquartile range
Counselling over the phone	216			
Previous experience with performing remote nutrition counselling				
Yes	83	38·8		
Very little	39	18·2		
No	92	43·0		
How would you compare phone nutrition counselling to usual nutrition counselling (face-to-face)?				
Superior to face-to-face counselling	7	3·3		
Similar to face-to-face counselling	53	25·2		
Inferior to face-to-face counselling	138	65·4		
Not certain	13	6·1		
Quality of counselling**				
Overall quality*			7·0	2·0
Technical quality*			7·0	4·0
Clinical quality*			7·0	2·0
Organisational difficulties*			7·0	3·0
Convenience†			4·0	2·0
Future use†			3·0	4·0
Perceived level of patient comfort in receiving remote counselling*			8·0	3·0
Types of difficulties reported during counselling				
Technical difficulties	69	56·2		
Interpersonal communication difficulties	18	14·6		
Difficulties due to lack of anthropometric measurements	31	25·2		
Difficulties/inconvenience from conducting the session in the home environment	3	2·4		
Other	2	1·6		
Perceived level of patient comfort in receiving remote counselling*			8·0	3·0
Perceived level of patient comfort in receiving remote counselling*			7·0	2·0
Types of difficulties reported during counselling				
Technical difficulties	90	46·9		
Interpersonal communication difficulties	37	19·3		
Difficulties due to lack of anthropometric measurements	54	28·1		
Difficulties/inconvenience from conducting the session in the home environment	4	2·1		
Other	7	3·6		
Counselling using an online platform	159			
Previous experience with performing online video nutrition counselling				
Yes	56	40·6		
Very little	20	14·5		
No	24	17·4		
I learned to use the online platforms during the coronavirus pandemic	38	27·5		
How would you compare online nutrition counselling to usual nutrition counselling (face-to-face)?				
Superior to face-to-face counselling	4	4·4		
Similar to face-to-face counselling	40	43·9		
Inferior to face-to-face counselling	44	48·4		
Not certain	3	3·3		
Quality of counselling**				
Overall quality*			8·0	2·0
Technical quality*			7·0	2·0
Clinical quality*			8·0	4·0
Organisational difficulties*			7·0	6·0
Convenience†			3·0	1·0
Future use†			3·0	1·0
Perceived level of patient comfort in receiving remote counselling*			7·0	2·0
Types of difficulties reported during counselling				
Technical difficulties	90	46·9		
Interpersonal communication difficulties	37	19·3		
Difficulties due to lack of anthropometric measurements	54	28·1		
Difficulties/inconvenience from conducting the session in the home environment	4	2·1		
Other	7	3·6		
Perceived level of client comfort in receiving remote counselling*			8·0	3·0
Types of difficulties reported during counselling				
Technical difficulties	69	56·2		
Interpersonal communication difficulties	18	14·6		
Difficulties due to lack of anthropometric measurements	31	25·2		
Difficulties/inconvenience from conducting the session in the home environment	3	2·4		
Other	2	1·6		

* Items scored on a ten-point Likert scale ranging from 1 to 10, where 1 = ‘very low’ to 10 ‘very high’.

† Items scored on a four-point response scale, where 1 = ‘very little’ and 4 = ‘high’.

Previous experience in providing online video counselling was indicated by 55·1 % of those who used online platforms, while 14·5 % indicated very little experience. Less than half (48·4 %) indicated that online consultation was inferior to a face-to-face consultation, while 43·9 % found the two technics similar. Only 4·4 % found online video counselling superior to face-to-face counselling. The median (interquartile range) score for overall quality among the study population was 8 (2). The technical quality score was 7 (2), and organisational difficulties 7 (3). The most frequent difficulties reported in using online counselling were technical difficulties (46·9 %), followed by a lack of anthropometric measurements (28·1 %), interpersonal or communication difficulties (19·3 %) and difficulties from conducting the session in the home environment (2·1 %).

Respondents who indicated using both phone and online platforms were asked to compare the two modalities. Almost half (49·4 %) indicated the quality is similar, 46·2 % reported higher quality of counselling using online video, whereas 4·3 % rated the quality of consultation while using the phone as higher.

### Associations between socio-demographic and occupational characteristics of dietitians and the overall quality of phone and online counselling

In a multivariate logistic regression with the dependent variable being the total quality score of counselling using the phone higher than 7, former experience in performing phone nutrition counselling was associated with a 2·5-fold increase in odds of a higher quality score (OR = 2·50, 95 % CI 1·38, 4·52, *P* = 0·02). Older age was also associated with a higher quality score (OR = 1·05, 95 % CI 1·01, 1·08, *P* = 0·005) (Table 4). In a multivariate logistic regression in which the dependent variable was the total quality score of counselling using online video platforms > 8, the only significant predictor variable was workload; specifically, respondents who continued full-time work had a five-fold increase in odds for a higher quality score (OR = 5·33, 95 % CI 1·091, 14·89, *P* = 0·001) (Table 5).

**Table 4:** Factors associated with a higher overall quality score using the phone for counselling in a multivariable logistic regression analysis*

	OR	95 % CI	*P*
Age (years)	1·05	1·01, 1·08	0·005
Academic degree (MSc and above *v*. BSc and below)	0·40	0·42, 1·41	0·40
Employment setting (public *v*. private)	1·00	0·51, 2·00	0·98
Workload during the pandemic (partial *v*. full)	0·96	0·49, 1·87	0·91
Previous experience using the phone in dietetic counselling (no experience *v*. experience)	2·50	1·39, 4·52	0·02
Constant	0·09		0·003

* Higher quality score was assigned a total quality score higher than the median value of seven points for performing telemedicine using the phone.

**Table 5:** Factors associated with a higher overall quality score using online video counselling in a multivariable logistic regression analysis*

	OR	95 % CI	*P*
Age (years)	0·99	0·94, 1·03	0·54
Academic degree (MSc and above *v*. BSc and below)	0·65	0·25, 1·69	0·38
Employment setting (public *v*. private)	1·11	0·41, 3·05	0·84
Workload during the pandemic (partial *v*. full)	5·33	1·91, 14·89	0·001
Previous experience using phone in dietetic counselling (no experience *v*. experience)	1·27	0·52, 3·10	0·61
Constant	0·17		0·13

* Higher quality score was assigned a total quality score higher than the median value of eight points for performing telemedicine using the phone.

## Discussion

Telemedicine has the potential to be an appropriate and effective tool for delivering healthcare to people with chronic conditions at a distance^(12)^. During the COVID-19 pandemic, telemedicine usage has increased rapidly in various clinical fields^(6–10,13)^, but the extent to which dietitians engaged in it and its overall quality is currently unknown. In the present study, we described the unique characteristics of nutrition consultation during the first phase of the COVID-19 pandemic in Israel. During the outbreak, the majority of dietitians reported a decrease in their work hours, and most of them performed mixed counselling by phone, online platforms or both methods. However, at least half of the dietitians implemented telemedicine without previous experience in consultation using these modalities. Former experience in performing nutritional counselling over the phone and older age were the only significant predictors of a higher quality score of counselling using the phone, while the only significant predictor for a higher quality score of counselling using online platforms was higher workload during the pandemic. Thus, it is possible that knowledge acquisition and training are the main keys to successful telemedicine implementation^(12)^.

The use of telemedicine holds potential advantages, including reducing healthcare costs, improving access to healthcare services and increasing patient compliance and outcomes^(12,14,15)^. Likewise, telemedicine can eliminate geographic barriers and facilitate international access to patients across the world^(14,15)^. Furthermore, telemedicine performed while using online platforms may also open unique opportunities to learn more about patients^(16)^. For instance, dietitians may ask to see the patient’s kitchen, food items, or serving dishes and conduct medication and supplementation reviews. They may also enable family members from separate locations to participate in the visit^(16)^. In the present study, the dietitians rated the overall quality of both phone and online consultation as relatively high. Nevertheless, at least half of the dietitians indicated that telemedicine is inferior to face-to-face consultation. Indeed, using telemedicine platforms may compel practitioners and patients to embrace a new norm that includes communicating with each other through video and audio^(17)^. However, it seems that telemedicine will not replace in-person consultations entirely, even though it can serve as a vital adjunct to patient care under certain circumstances^(15)^. Barriers to receiving telemedicine, as reported by patients, include lack of access to the infrastructure required, feeling uncomfortable with the technology, security concerns or desire to be seen in-person by their practitioners^(15,17)^. Thus, the implementation of telemedicine can be a challenge, and some questions remain about the acceptability of it by patients and practitioners^(12)^. Indeed, some of these challenges were raised by the dietitians in the present study; the most prevalent reported difficulties for either phone or online platforms were technical difficulties, lack of anthropometric measurements and interpersonal communication difficulties. In order to increase telemedicine success, some instructions have been suggested including minimising ambient sounds, speaking clearly, making pauses frequently to address patient questions and, in case of online platform usage, keeping the camera directly in front of the practitioner’s face at eye level, and dressing professionally^(14)^. Moreover, comfort level and general health literacy are important factors that must be considered in strategies to increase telemedicine utilisation^(14)^. Additional important tools that might improve telemedicine services by a dietitian are also health devices such as smartwatches^(14)^, electronic scales^(18)^ and continuous glucose monitoring^(19)^. However, maintaining patient privacy should be considered when using electronic data transfer^(15)^.

During the outbreak, differences in the type of referrals were noted among more than half of the respondents in comparison to the usual. The majority of dietitians identified an increase in referrals for weight loss or weight maintenance, and this is not surprising as obesity and its comorbidities were found to be risk factors for COVID-19-related morbidity and mortality^(20)^. Moreover, the necessary lockdown imposed in order to mitigate the spread of COVID-19 may increase the risk of obesity and its health consequences^(20)^. This is due to irregular eating patterns, poor diet quality, inadequate physical activity, excess screen time, disrupted sleep routines, higher stress levels and social immobility, which were probably evolved or exacerbated during the outbreak, at least in some populations^(1,19,20)^.

The present study reports on the perception of dietitians regarding the utilisation of telephone and video consultation platforms during the COVID-19 outbreak. While our study emphasises the potential benefits of telemedicine, especially in light of potential crisis-induced lockdown, additional tools and training are needed to optimise remote nutrition counselling. At the same time, the present study has several limitations. First, the data are cross-sectional, and as such, causality cannot be inferred. Second, we have used a convenience sample. Moreover, it is possible that those who responded to the survey were those who are also more oriented to telemedicine platform usage. Therefore, the generalisability of the study results is limited. Although it is difficult to predict how the post-COVID-19 period will impact the health system, it seems that current circumstances have encouraged more dietitians to set up telemedicine as part of their practice. Future observational studies should re-examine the uptake of online counselling among dietitians, while intervention studies should explore the use of telemedicine in various nutrition fields and target populations in order to assess their effectiveness.

## Conclusions

Our findings suggest that telemedicine holds a promising potential for nutritional consultation during a pandemic outbreak period. Data are needed for establishing adequate standards of nutritional care and practice using telemedicine in crisis as well as in routine periods.

## References

[1] Naja F & Hamadeh R (2020) Nutrition amid the COVID-19 pandemic: a multi-level framework for action. Eur J Clin Nutr 74, 1117–1121. 32313188 10.1038/s41430-020-0634-3 PMC7167535

[2] Laviano A , Koverech A & Zanetti M (2020) Nutrition support in the time of SARS-CoV-2 (COVID-19). Nutrition 74, 110834. 32276799 10.1016/j.nut.2020.110834 PMC7132492

[3] Fruhbeck G , Baker JL , Busetto L et al. (2020) European association for the study of obesity position statement on the global COVID-19 pandemic. Obes Facts 13, 292–296. 32340020 10.1159/000508082 PMC7250342

[4] Calder PC , Carr AC , Gombart AF et al. (2020) Optimal nutritional status for a well-functioning immune system is an important factor to protect against viral infections. Nutrients 12, 1181. 10.3390/nu12041181 PMC7230749 32340216

[5] Clarfield AM , Dwolatzky T , Brill S et al. (2020) Israel Ad Hoc COVID-19 committee: guidelines for care of older persons during a pandemic. J Am Geriatr Soc 11, 16554. 10.1111/jgs.16554 PMC7272988 32392624

[6] Saleem SM , Pasquale LR , Sidoti PA et al. (2020) Virtual ophthalmology: telemedicine in a COVID-19 Era. Am J Ophthalmol 216, 237–242. 32360862 10.1016/j.ajo.2020.04.029 PMC7191296

[7] Prasad A , Brewster R , Newman JG et al. (2020) Optimizing your telemedicine visit during the COVID-19 pandemic: practice guidelines for patients with head and neck cancer. Head Neck 42, 1317–1321. 32343458 10.1002/hed.26197 PMC7267295

[8] Grossman SN , Han SC , Balcer LJ et al. (2020) Rapid implementation of virtual neurology in response to the COVID-19 pandemic. Neurology 94, 1077–1087. 32358217 10.1212/WNL.0000000000009677

[9] Compton M , Soper M , Reilly B et al. (2020) A feasibility study of urgent implementation of cystic fibrosis multidisciplinary telemedicine clinic in the face of COVID-19 pandemic: single-center experience. Telemed J E Health 26, 978–984. 32357084 10.1089/tmj.2020.0091

[10] Tanaka MJ , Oh LS , Martin SD et al. (2020) Telemedicine in the era of COVID-19: the virtual orthopaedic examination. J Bone Joint Surg Am 17, e57. 10.2106/JBJS.20.00609 PMC7224627 32341311

[11] Vidal-Alaball J , Flores Mateo G , Garcia Domingo JL et al. (2020) Validation of a short questionnaire to assess healthcare professionals’ perceptions of asynchronous telemedicine services: the Catalan version of the health optimum telemedicine acceptance questionnaire. Int J Environ Res Public Health 17, 2202. 10.3390/ijerph17072202 PMC7178015 32218310

[12] Flodgren G , Rachas A , Farmer AJ et al. (2015) Interactive telemedicine: effects on professional practice and health care outcomes. Cochrane Database Syst Rev. Published online: 07 September 2015. doi: 10.1002/14651858.CD002098. PMC6473731 26343551

[13] Siniscalchi M , Zingone F , Savarino EV et al. (2020) COVID-19 pandemic perception in adults with celiac disease: an impulse to implement the use of telemedicine: COVID-19 and CeD. Dig Liver Dis 52, 1071–1075. 32425731 10.1016/j.dld.2020.05.014 PMC7229921

[14] Contreras CM , Metzger GA , Beane JD et al. (2020) Telemedicine: patient-provider clinical engagement during the COVID-19 pandemic and beyond. J Gastrointest Surg 24, 1692–1697. 32385614 10.1007/s11605-020-04623-5 PMC7206900

[15] Blue R , Yang AI , Zhou C et al. (2020) Telemedicine in the era of COVID-19: a neurosurgical perspective. World Neurosurg 139, 549–557. 32426065 10.1016/j.wneu.2020.05.066 PMC7229725

[16] Calton B , Abedini N & Fratkin M (2020) Telemedicine in the time of coronavirus. J Pain Symptom Manage 60, e12–e14. 10.1016/j.jpainsymman.2020.03.019 PMC7271287 32240756

[17] Mann DM , Chen J , Chunara R et al. (2020) COVID-19 transforms health care through telemedicine: evidence from the field. J Am Med Inform Assoc 27, 1132–1135. 32324855 10.1093/jamia/ocaa072 PMC7188161

[18] Krukowski RA & Ross KM (2020) Measuring weight with electronic scales in clinical and research settings during the coronavirus disease 2019 pandemic. Obesity 28, 1182–1183. 32339394 10.1002/oby.22851 PMC7267353

[19] Singh AK , Gupta R , Ghosh A et al. (2020) Diabetes in COVID-19: prevalence, pathophysiology, prognosis and practical considerations. Diabetes Metab Syndr 14, 303–310. 32298981 10.1016/j.dsx.2020.04.004 PMC7195120

[20] Woo Baidal JA , Chang J , Hulse E et al. (2019) Zooming toward a telehealth solution for vulnerable children with obesity during coronavirus disease. Obesity 27, 1184–1186. 32352650 10.1002/oby.22860 PMC7267304

